# Optimization of Forward Osmosis for Oil Refinery Effluent Desalination Using Response Surface Methodology

**DOI:** 10.3390/membranes16030086

**Published:** 2026-02-28

**Authors:** Elorm Obotey Ezugbe, Sudesh Rathilal, Emmanuel Kweinor Tetteh

**Affiliations:** Green Engineering and Sustainability Research Group, Department of Chemical Engineering, Faculty of Engineering and The Built Environment, Durban University of Technology, Durban 4001, South Africa

**Keywords:** forward osmosis, permeate flux, optimization, response surface methodology (RSM)

## Abstract

Repurposing usage of oil refinery wastewater with retrofitted desalination technology necessitates the optimization of a forward osmosis (FO) technology. Herein, factors such as draw solution concentration (DS-C) and feed and draw solution flow rates (FS-FR, DS-FR) play significant roles. In this study, the individualistic and interaction effects of these factors were explored to ascertain the FO performance. The effects of these operating factors, DS-C (20–50 g/L), DS-FR (7.5–9.4 L/h), and FS-FR (7.5–9.4 L/h), and their interactive effects on the permeation flux and rejection of Cl^−^, SO_4_^2−^ and CO_3_^2−^ from oil refinery effluent, were studied using the Box–Behnken design (BBD) of response surface methodology (RSM). Statistical models were developed to optimize the operating conditions. The analysis of variance and the developed response models were used to evaluate the data at a 95% confidence level. Three confirmatory runs were conducted based on the optimum conditions (FS-FR: 9.2 L/h; DS-FR: 9.4 L/h; DS-C: 32.6 g/L). At a desirability of 81%, average rejections of 94.59 ± 0.32% for CO_3_^2−^ and 100% for SO_4_^2−^ were obtained. Average Cl^−^ enrichment was 35.5 ± 5.15% and average permeation flux of 3.64 ± 0.13 L/m^2^ h were achieved, suggesting that RSM was a suitable tool for optimizing FO for desalinating the effluent. In addition, the average recovered permeation flux of 86.01 ± 2.66% demonstrated the effectiveness of the FO membrane after cleaning.

## 1. Introduction

The water–energy nexus provides a perspective of holistic management of water and energy resources, as these two resources interdepend on each other [1]. This proves the necessity with which energy efficiency in the use of water and the subsequent treatment of water and wastewater should be pursued. Much research is still ongoing to improve the use of energy in water and wastewater treatment processes. For example, seawater desalination has seen a great shift from the use of the conventional thermal processes like vapor compression, multi-effect distillation and multi-stage flash systems to the use of relatively less energy-intensive reverse osmosis (RO) processes [2]. In the Jeddah desalination plant, Saudi Arabia, it was observed that a shift from conventional thermal desalination to the use of RO reduced the cost of seawater desalination to below $0.5/m^3^ as well as energy use from 6.1 kWh/m^3^ to 2.0–3.0 kWh/m^3^ [3].

In oil refinery effluent (ORE) treatment, many methods are used, spanning from pretreatment to advance treatment to make the effluent fit for disposal or reuse. Due to the high concentrations of residual oils in the effluent, pretreatment processes are mainly targeted towards the removal or recovery of residual oils. Commonly used pretreatment processes include gravity separation followed by skimming. This process allows for the separation of oil, water, and other suspended materials through the settling of the heavier components freely under gravity based on their specific weights. The API oil–water separator is most often used in this process [4,5]. Intermediate ORE treatment processes mainly employed include dissolved air flotation (DAF), coagulation and flocculation, and biological treatment. These treatment methods further reduce the residual oil component, suspended materials, and the refractory components of the ORE [6]. In advanced treatment methods, membrane technology and advanced oxidation processes (Fenton reaction, photo-Fenton, ozone/UV, TiO_2_/UV, etc.) have been applied extensively in ORE treatment [5,7].

AOPs specialize in oxidizing the organic components of ORE into more stable products like CO_2_ and H_2_O. During this process, hydroxyl radicals (OH*) or other similar reactive species like sulphate radical anion (SO_4_*^−^) are generated either through the application of an appropriate catalyst or the use of ultraviolet (UV) light [8], which then attack the organic components of the effluent. AOPs are, however, limited in their application, as they are unable to degrade inorganic components of ORE.

Membrane technology has a proven history of efficient treatment of wide variety of effluents including ORE [9]. The application of pressure-based membrane processes, such as RO and NF, in ORE treatment and desalination is well established. Other forms of membrane processes such as electrodialysis, membrane distillation, and FO are still under extensive research for their applications in this area [10,11]. While RO and other pressure-driven membrane processes, such as nanofiltration (NF) and ultrafiltration (UF), may have improved water treatment and desalination, their dependence on external hydraulic pressure poses significant concerns regarding energy utilisation. Among the possible solutions to this problem is the application of forward osmosis (FO) in various capacities, including as a pretreatment process for seawater desalination by RO.

FO depends on the osmotic pressure gradient between two solutions to cause the movement of water across a semipermeable membrane. The prospects associated with this less energy intensive process has led to a wide range of research on its application for different purposes, including dewatering [12,13,14], concentration of wastewater streams [15,16], FO hybrid systems for wastewater treatment and desalination [17,18,19], resource recovery [20,21], etc.

Many studies have examined FO to determine the process variables and their effects on process efficiency. In a study to investigate the impacts of DS-C on FO performance, Xu, et al. [22] observed that even though permeate flux increased with DS-C, flux behavior was non-linear, possibly due to internal concentration polarization and dilution DS by permeate. The authors also noted that feed flow rate had limited effects on permeate flux. Zhao and Zou [23] studied the effects of working temperature on the separation performance and membrane cleaning of FO. The authors observed that at higher temperatures, there is a higher initial flux and a higher concentration factor. However, this adversely affected the cleaning process, resulting in severe membrane scaling.

In other studies on this subject, Zhang, et al. [24] investigated the effects of reverse solute diffusion (RSD) on scaling, in which the authors showed that while the presence of Ca^2+^ in DS promotes scaling, this effect is only noticed at points where the specific RSD (ratio of Ca^2+^ flux to water flux) was greater than the actual Ca^2+^ concentration in the FS. The impact of membrane orientation on the performance of FO was investigated by Hawari, et al. [25]. From their results, it was observed that when the membrane’s active layer faces the FS (known as the FO mode), permeation flux declines due to the effects of dilutive internal concentration polarization. The impacts of air sparging on fouling and concentration polarization in FO was studied by Ferrari, et al. [26]. The submerged FO membrane was found to maintain high water flux and low fouling due to continuous air sparging. Xiao, et al. [27] investigated the effects of pH, FS and DS properties on membrane flux and fouling using alginate as model organic matter. The results showed that with increasing organic loading, water flux decreased, and fouling resistance induced by the pH of the FS also decreased with increasing pH.

It is apparent that several studies have focused primarily on the individual effects of FO process variables on process performance and efficiency. This one-factor-at-a-time (OFAT) approach is time consuming and expensive to run. In addition, having identified FO as a potential energy-saving desalination and wastewater treatment technique, it is essential to consider cross-factor interactions and their effects on the FO process. This will optimize and maximize the performance of the FO process.

With the current study in focus, the Box–Behnken design (BBD) of response surface methodology (RSM) was used to optimize FO for desalination of a local South African waste oil refinery effluent. The operating factors of interest were feed solution flow rate (FS-FR), draw solution flow rate (DS-FR), and draw solution concentration (DS-C). These factors were chosen based on the observation of our previous study [28], in which the individual impacts of these factors were established. The pronouncement of RSD was particularly of interest, and the possibility of reducing the RSD at the same time, achieving good permeation flux and salt rejection, encouraged the optimization study. In addition, oil refinery effluents pose significant environmental concerns when discharged without adequate treatment. The negative impacts of Cl^−^, SO_4_^2−^, and CO_3_^2−^ salts on the environment and downstream water treatment channels are well known. Again, to maximise the potential of the FO process for FO-RO applications in water recovery and reuse, process optimisation is necessary. There are other optimisation tools like Taguchi’s technique and the orthogonal methodology. RSM enables the study of the effects of multiple factors and their interactions across different levels. RSM provides the best experimental conditions with a lower number of experimental runs and surface plots to visualise the cross-factor interactions [14,29,30].

Furthermore, the study’s specific contribution to forward osmosis research is limited to optimising a laboratory-scale FO system treating real oil refinery effluent, a highly variable and fouling-prone industrial stream that has received insufficient attention in optimisation studies. The novelty lies in integrating hydrodynamic conditions—feed-solution flow rate (FS-FR) and draw-solution flow rate (DS-FR)—with draw-solution concentration within a Box–Behnken response surface framework to simultaneously evaluate water flux, chloride enrichment, and the rejection behaviour of key divalent ions. This approach provides a comprehensive assessment of process behaviour under realistic operating conditions and offers practical insights for enhancing FO performance in industrial wastewater desalination applications.

## 2. Materials and Methods

The experimental cross flow FO system employed in this study is similar to that described in our previous studies [31]. The flat-sheet cellulose triacetate (CTA) membrane used in this study was embedded in a polymer mesh support (Sterlitech, Auburn, WA, USA). Additional specifications, including thickness, pore size and temperature, were 0.09652 mm, 0.307 ± 0.003 nm, and 60 °C, respectively. The feed solution (synthetic ORE) was prepared according to procedures as used by [28]. After the optimisation study, real ORE (sampled from the sewer of the effluent treatment plant of a local South African waste oil treatment plant. This was described in our previous study [28], where we used for confirmatory runs to validate the generated models further. Table 1 shows the characteristics of the feed.

### 2.1. Box–Behnken Design (BBD)

BBD is one of the response-surface designs widely used, alongside the central composite design (CCD), in experimental design. By applying BBD, the number of experimental runs that should be conducted is optimized in such a way that it analyses the interactions that are possible among the studied factors and among their potential impacts on the response of the process [32].

By utilising the BBD (Design Expert software V.11.1.0.1, Stat-Ease Inc., Minneapolis, MA, USA), 15 experimental runs were generated, including three replications. The three different normalised central levels were coded as −1, 0, and +1, corresponding to the minimum, central point, and maximum for the factors considered. These experimental runs were randomised to eliminate bias. The optimal conditions were used for confirmatory runs, comprising three independent runs at the same level.

### 2.2. Process Description

**Figure 1:**
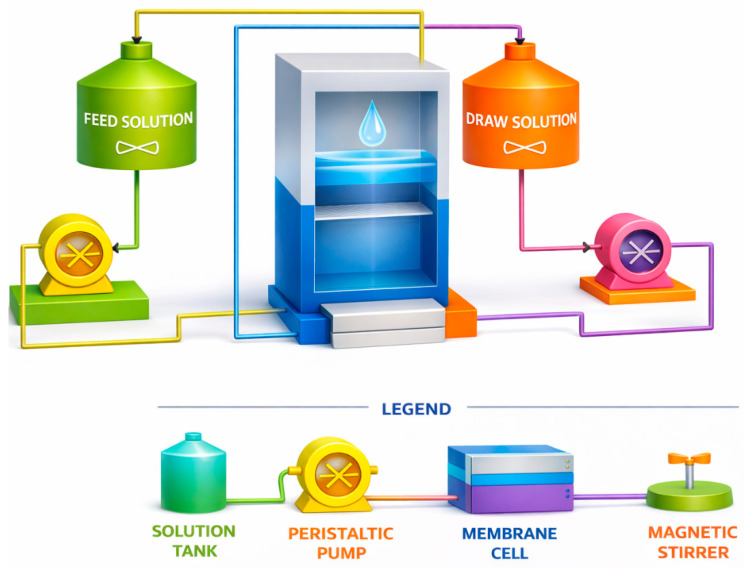
Process flow diagram showing the main equipment used in the experiment; adapted from [28].

According to the experimental design, three factors (DS concentration, DS cross-flow (DS-FR) rate, and FS cross-flow (FS-FR) rate) were varied, and their interactive effects on permeation flux and salt rejection were analysed. DS concentration varied at 20, 35 and 50 g/L NaCl. DS-CFR was varied at 7.5, 8.4, and 9.4 L/h, and FS-CFR was varied at the same values. The DS configuration adopted was the continuous dilution method, where the draw solution was diluted with the permeate water for the entire duration of the experiment [26]. The membrane was oriented with the active layer facing the feed solution. Counter-current flow of DS and FS was used in this study [33,34]. For each run, the FS tank was filled to the 3 L mark, and the DS tank to the 1 L mark. Each experiment lasted for 6 h. Figure 1 shows the process flow diagram.

Since the permeate diluted the DS, the dilution factor (*Df*), component rejection (CJ), and Permeate flux (J) were calculated using Equations (1), (2), and (3), respectively.

(1) $$\mathrm{Dilution}\;\mathrm{factor}\;(\mathrm{Df})=\frac{V_{f,DS}}{V_{p}}$$

where *V_f,DS_* is the final volume of the *DS* and *V_p_* is the volume of permeate.

(2) $$\mathrm{Component}\;\mathrm{Rejection}\;(\mathrm{CJ})\;(\%)=\frac{C_{0}-{D_{f}C}_{f}}{C_{0}}\times 100$$

where *C*_0_ and *C_f_* are initial and final concentrations of the targeted component in the FS and DS, respectively, and *Df* is the dilution factor.

(3) $$\mathrm{Permeate}\;\mathrm{flux}\;(J)=\frac{Volume\;of\;permeate\;(L)}{Effective\;membrane\;area\;\left(m^{2}\right)\times time\;(h)}$$

The volume of permeate was determined by subtracting the initial volume of the draw solution from the final volume.

### 2.3. Mass Transport in FO Membranes

**Figure 2:**
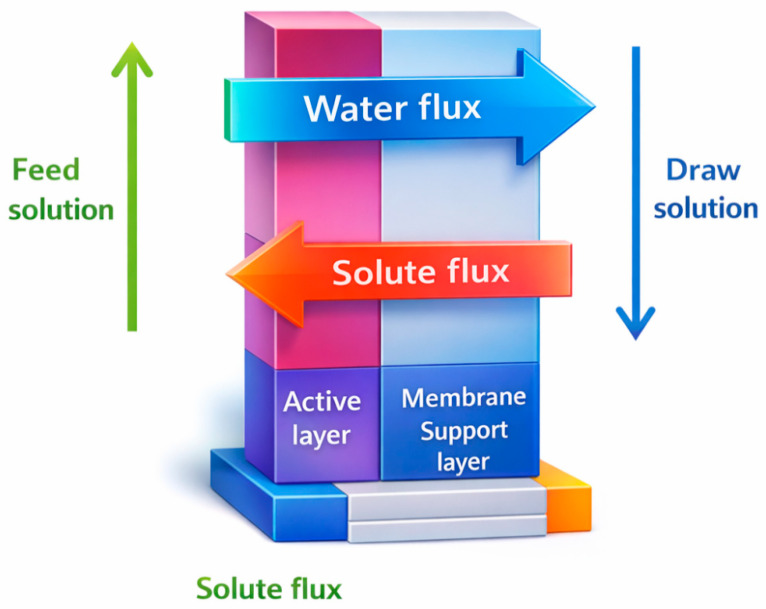
Schematic diagram of the mass transport across the FO membrane. Modified from Roest [36].

The movement of materials across the FO membrane can be categorized into two forms, namely solvent transport and solute transport. As illustrated in Figure 2, the transport of these components primarily depends on the intrinsic properties of the membrane, such as water permeability designated as A, solute permeability B, and the structural parameter of the support layer, S, of the membrane [35].

Solvent (water) transport through the semipermeable membrane is generally governed by Equation (4)

(4) $$J_{w}=A\left(∆\pi -∆P\right)$$

where $J_{w}$ is water flux (L/m^2^·h—LMH), A is the water permeability coefficient of the membrane (LMH/bar), ∆π is the osmotic pressure differential in bar across the membrane, and ∆P is the hydraulic pressure differential in bar across the membrane. In FO, ∆P = 0, hence the dependence of $J_{w}$ on ∆π. In RO, ∆P > ∆π, hence the dependence of $J_{w}$ on ∆P.

Solute transport across the FO semipermeable membrane is bidirectional. Solute diffuses across the membrane simultaneously in both directions [37]. This solute diffusion is generally governed by Fick’s law of diffusion (5);

(5) $$J_{s}=B\left(∆c\right)$$

where $J_{s}$ is the flux (g/m^2^·h) of an individual species of salt diffusing through a semipermeable membrane, *B* is solute permeability coefficient (m/s), and ∆*c* is the trans-membrane concentration differential [37,38].

## 3. Results and Discussion

Table 2 shows the design matrix and the results obtained after the experimental runs. These include the coded study factors, the actual results, and the model-predicted results for the response factors. The exact values are the measured response data from a specific run, whereas the predicted values are computed from the model. Statistical robustness was enhanced by reporting standard deviations for all responses and providing 95% confidence intervals for experimental means and model predictions. The experimental matrix in Table 2 identifies centre-point replicates (Runs 3, 13, and 14) used to estimate pure error and assess curvature within the Box–Behnken design. Reproducibility was further evaluated by confirming runs at the predicted optimum and by replicating runs under edge conditions. Measurement uncertainty, error propagation, and residual diagnostics is presented in terms of the actual-versus-predicted behaviour and lack-of-fit assessments (ANOVAA), which strengthened model adequacy and overall interpretability.

### 3.1. Model Fitting and Statistical Analysis

Equations (6)–(9) represent the models generated by the software for the various responses in coded form. These are second order quadratic models in their reduced form, expressed as functions of the input and output variables. Model reduction was necessary in order to improve the predictability of the response variables.

Flux = 3.21 − 0.3981A + 0.1482B + 0.7133C − 0.337AB − 0.13AC − 0.5217BC − 0.4939C^2^ (6)

Cl^−^ enrichment = 27.01 − 0.4338A − 4.94B + 17.4C − 6.2AB + 13.05AC + 5.08B^2^ + 8.19C^2^ (7)

SO_4_^2−^ rejection = 98.84 − 2.87A − 1.85B + 1.71C − 3.75AB + 1.48AC − 2.83A^2^ (8)

CO_3_^2−^ rejection = 94.95 − 0.6162A + 1.25B + 1.88C − 1.04AB + 0.405AC − 3.22BC − 1.63A^2^ − 1.47C^2^ (9)

The extent to which terms of the models can affect the response are associated with the positive and negative coefficients of the terms. Negative coefficients indicate unfavourable effects of the factors, whereas positive coefficients indicate that a factor or combination of factors contributes favourably to contaminant removal. Again, the magnitudes of the coefficients correlate with the degree to which the response variable is affected [39]. To this effect, the impact of the model terms of each model in an ascending order are as follows: flux = C > B > AC > AB > A > C^2^ > BC; Cl^−^ enrichment = C > AC > C^2^ > B^2^ > A > B > AB; SO_4_^2−^ rejection = C > AC > B > A^2^ > A > AB; CO_3_^2−^ rejection = C > B > AC > A > AB > C^2^ > BC. A, B, and C are the individual factors; AB, AC, and BC denote the interactions between the factors; and A2, B2, and C2 represent the quadratic effects, in which each factor interacts with itself.

The statistical relevance and accuracy of the models were verified by the analysis of variance (ANOVA), which is a powerful statistical tool used to explain the variations in the magnitude of a response variable of interest based on Equation (10), which is the second-order polynomial Equation [40,41].

(10) $$Y=\beta_{0}\sum_{i=1}^{k}\beta_{i}x_{i}+\sum_{i=1}^{k}\beta_{ii}x_{i}^{2}+\sum_{i=1}^{k-1}+\sum_{j=2}^{k}\beta_{ij}x_{i}x_{j}+\varepsilon \;$$

where Y is the response variable, β_0_ is a constant, β_i_ is the coefficient of regression, k is the number of independent variables (in this case 3, which are within the range of −1 to +1 in coded form) and ε is the unknown error constant. The coded values were express by the following Equation (11);

(11) $$x_{i}=\frac{X_{i}-X_{i0}}{∆X_{i}};i=1,\;2,\;3,\ldots ..\;k$$

where x_i_ represents the coded values and X_i_ is the real values of the independent variables, X_i0_ = real values in the center plane and ∆X_i_ = step change.

The overall efficiency of the model’s prediction and prediction variation is indicated by the coefficient of determination (R^2^), which is calculated according to Equation (12).

(12) $$R^{2}=1-\frac{{SS}_{residual\;error}}{{SS}_{total}}$$

This coefficient is used to quantify the variation in predicted responses relative to the mean response [42]. For good model prediction efficiency, R^2^ should be close to 1. In addition, adjusted R^2^ and predicted R^2^ quantify prediction efficiency. The difference between these two values should be less than 0.2 for any statistically relevant model. Table 3, Table 4, Table 5 and Table 6 present the ANOVA results for all generated models.

**Figure 3:**
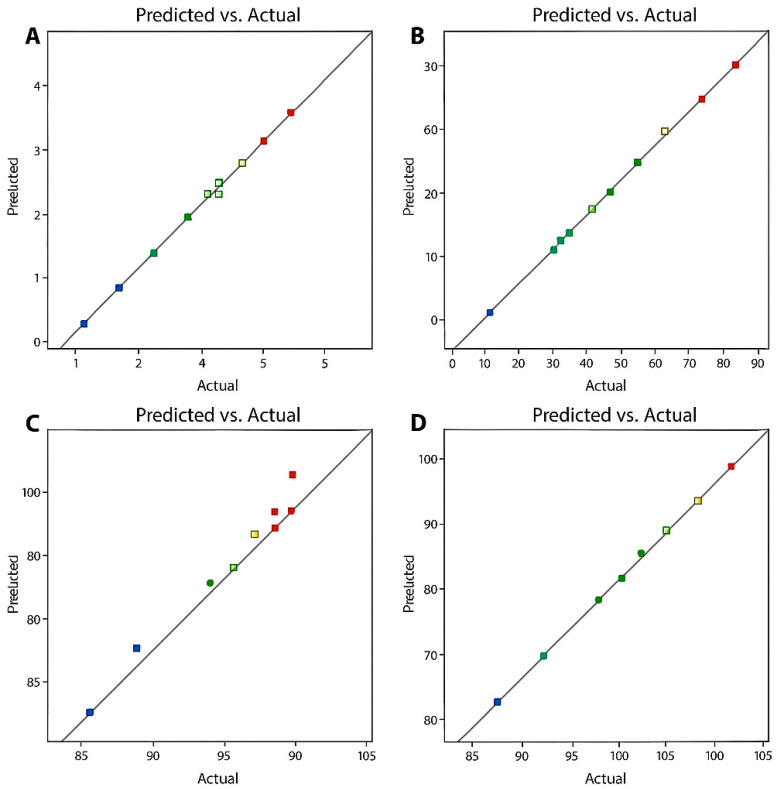
Predicted vs. actual values for (**A**): permeation flux, (**B**): Cl^−^ enrichment, (**C**): SO_4_^2−^ rejection and (**D**): CO_3_^2−^ rejection.

From Table 3, Table 4, Table 5 and Table 6, it can be seen that the R^2^ values of 0.9901, 0.9967, 0.9307, and 0.9919, respectively are significantly close to 1, indicating the validity of the generated quadratic models. Again, in each case, it can be seen that the adjusted R^2^ (0.9802, 0.9934, 0.8788 and 0.9812) and the Predicted R^2^ (0.9639, 0.9759, 0.8047 and 0.9329) are in reasonable agreement with each other, that is, having a difference of less than 0.2. Furthermore, the adequate precision for all models exceeds 4. This value measures the signal-to-noise ratio. Values greater than 4 indicate an adequate model, which can be safely used to navigate the entire design space. Also, as shown in Figure 3, the data fit a straight line. This indicates the validity of the generated quadratic models [43]. The lack-of-fit (LOF) in all cases was not significant. This is good for the validity of the models, as the significance of LOF indicates the inability of the model to sufficiently describe the functional agreements between the experimental factors and the response variables. All other model accuracy indicators, such asthe Fisher variation ratio (F-values) and probability (*p*-values), were all within the limits that indicate the significance of a model [30].

### 3.2. Effects of Independent Process Variables on FO Performance

The independent variables for this study were FS-FR, DS-FR and DS-C. Extensive study of the individual effects of these variables on the responses (flux, Cl^−^ enrichment, SO_4_^2−^ rejection and CO_3_^2−^ rejection) can be found in the authors’ previous study [28]. To ascertain the individual effects of the process variables, each variable was varied while keeping the other two at their midpoint values.

#### 3.2.1. Effects of Process Variables on Permeation Flux

**Figure 4:**
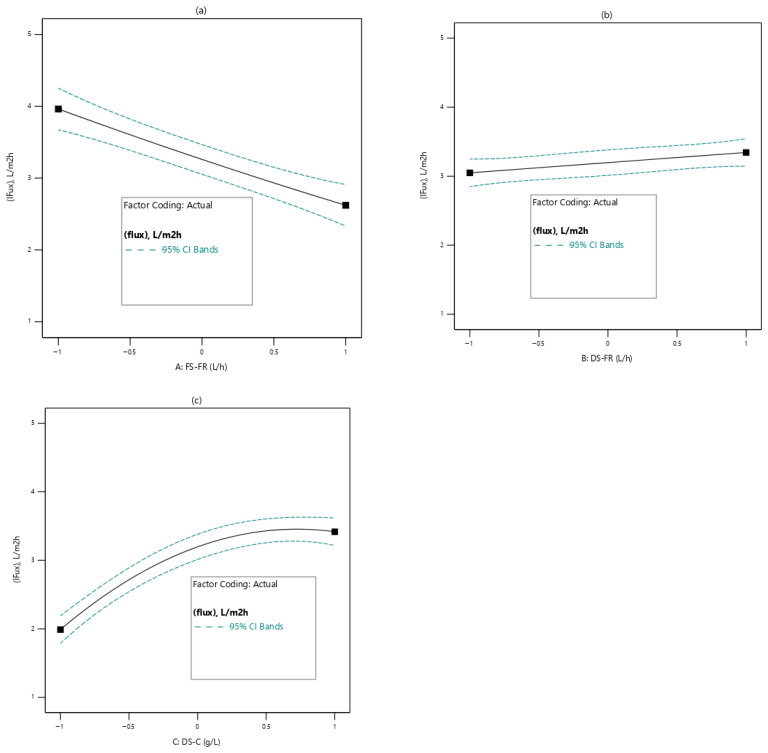
Effects of process variables on permeation flux; (**a**): FS-FR; (**b**): DS-FR; (**c**): DS-C.

Figure 4 shows the effects of the various process variables on permeation flux. From Figure 4a, the variation of FS-FR with permeation flux is shown. The linear relationship indicates that permeation flux decreases as FS-FR increases. This may be due to the following reason: because FO is a dilution process, there is a need for sufficient contact between the FS and the DS to facilitate the movement of water molecules from the FS to the DS. As FS-FR decreases, the dilution factor increases, leading to a higher rate of water-molecule transport through the membrane into the DS. Consequently, the net driving force from the DS becomes reduced, leading to low flux, where similar trends have been reported [28]. From Figure 4b, the relationship between permeation flux and DS-C is shown. The linear relationship indicates that permeation flux increases with DS-FR. This may be due to ECP’s influence on the DS side of the membrane. The creation of turbulence at the DS-membrane boundary layer at increased DS-FR may have reduced the impact of ECP, thereby allowing greater permeate transport [44]. Figure 4c shows the effects of DS-C on permeation flux. The relationship is nonlinear, tapering towards a plateau. Increasing DS-C increases the permeation flux. The DS-C provides the driving force for water transport through the membrane. However, a continual increase in DS-C also increases reverse solute flux, which tends to balance the osmotic gradient between the FS and the DS. As the system approaches equilibrium, water transport becomes constant [45].

#### 3.2.2. Effects of Process Variables on Cl^−^ Enrichment of FS

**Figure 5:**
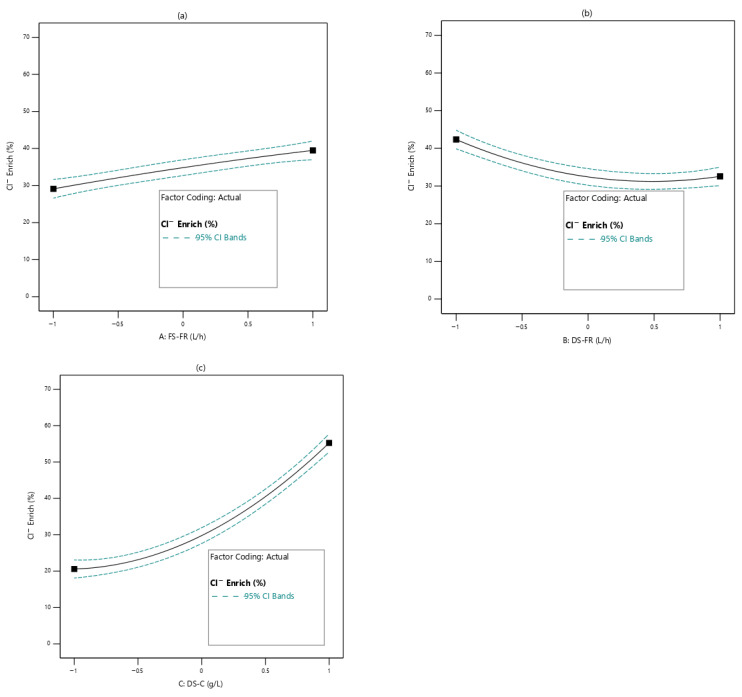
Effects of process variables on Cl^−^ enrichment of FS; (**a**): FS-FR; (**b**): DS-FR; (**c**): DS-C.

The feed solution was enriched with Cl^−^. This implies that the Cl^−^ concentration in the feed increased after the FO process. Cl^−^ enrichment, described as reverse solute diffusion, results from the backward movement of the Cl^−^ due to the difference in concentrations of Cl^−^ between the FS and the DS. Figure 5 shows the effects of the process variables on Cl^−^ enrichment of the FS. From Figure 5a, the influence of FS-FR is presented. Varying the FS-FR has little influence on Cl^−^ enrichment of the FS. In Figure 5b, the effect of DS-FR is presented. As DS-FR increases, Cl^−^ enrichment of the FS decreases, but rather weakly. This could be due to the association of high flow rates with turbulence at the membrane–solution boundary layer. This turbulence sweeps away the accumulated solutes that tend to diffuse through the membrane. Hence, as DS-FR increases, the Cl^−^ enrichment of the FS reduces [46]. The DS-C, by far, showed the most influence on the Cl^−^ enrichment of the FS (Figure 5c). The DS-C, being higher in Cl^−^ concentration than the FS, provides a concentration gradient along which Cl^−^ moves into the FS. The higher the Cl^−^ concentration of the DS, the greater is the backwards diffusion of the Cl^−^ into the FS. This accounts for the trend shown in Figure 5c [24].

#### 3.2.3. Effects of Process Variables on SO_4_^2−^ and CO_3_^2−^ Rejection

**Figure 6:**
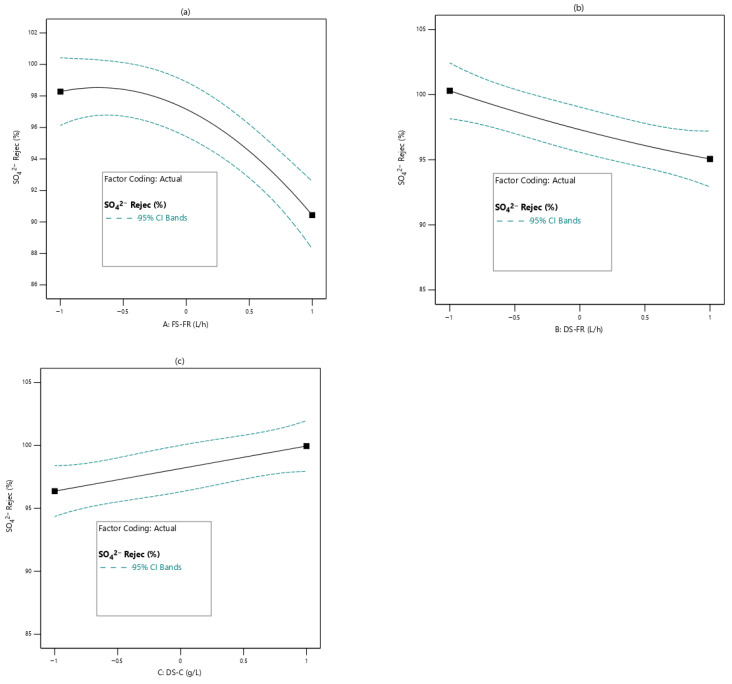
Effects of process variables on SO_4_^2−^ rejection efficiency (**a**): FS-FR; (**b**): DS-FR; (**c**): DS-C.

**Figure 7:**
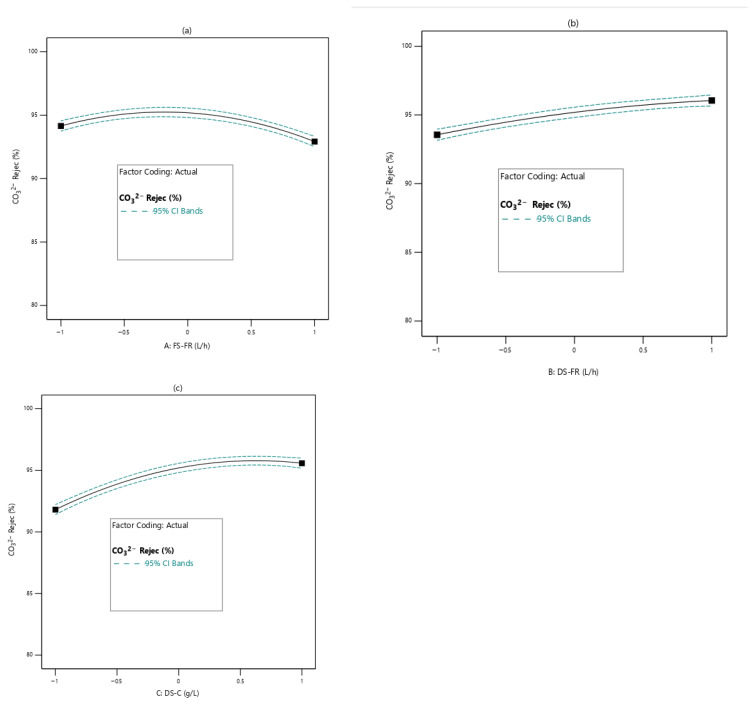
Effects of process variables on CO_3_^2−^ rejection efficiency; (**a**): FS-FR; (**b**): DS-FR; (**c**): DS-C.

Figure 6 and Figure 7 respectively show the effects of the process variables on SO_4_^2−^ and CO_3_^2−^ rejection efficiency. Increase in FS-FR shows an overall decline in SO_4_^2−^ and CO_3_^2−^ rejection (Figure 6a and Figure 7a) with SO_4_^2−^ rejection showing a steeper decline. For DS-FR, SO_4_^2−^ and CO_3_^2−^ rejection exhibit opposite trends. As DS-FR increases, SO_4_^2−^ rejection decreases while CO_3_^2−^ rejection increases (Figure 6b and Figure 7b). With DS-C, both SO_4_^2−^ and CO_3_^2−^ rejection show an increasing trend with an increase in DS-C (Figure 6c and Figure 7c); the relation for SO_4_^2−^ rejection is linear, while that of CO_3_^2−^ rejection increases and tapers to a plateau. To explain the effects of DS-C on rejection ion (Figure 6c and Figure 7c), the bidirectional transport of ions through the membrane pores must be considered. Ions move through the same pores that water permeates. At higher DS-C, more water is drawn through the membrane pores, whereas the movement of SO_4_^2−^ and CO_3_^2−^ is limited. In addition, at increased DS-C, the reverse diffusion of draw solutes becomes more pronounced. This implies that the membrane pores at any point in time will not be easily permeable to the SO_4_^2−^ and CO_3_^2−^, which happen to be large in size due to their divalent nature.

### 3.3. Effect of Process Variables Interaction on FO Performance

**Figure 8:**
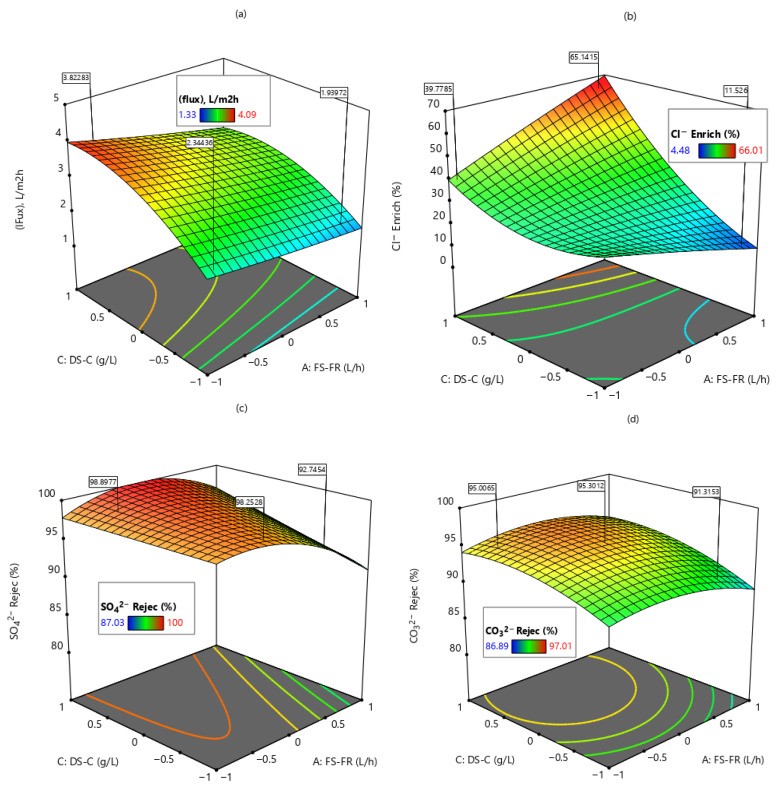
Response surface plots showing the interactive effects of DS-C and FS-FR for: (**a**) permeation flux and (**b**) Cl^−^ enrichment, (**c**) SO_4_^2−^ rejection and (**d**) CO_3_^2−^ rejection.

The cross-factor interactions presented in Figure 8 represent the interactions between the two most significant factors affecting the various responses, as identified by the generated models. Response surface plots are graphical representations of the relationship between two independent variables and a response. These two independent variables are plotted on the x and y axes, while the response is plotted on the z-axis.

From Figure 8, the most significant factors affecting the response variables are FS-FR and DS-C (AC). To study their interactive effects, the third factor (DS-FR) was fixed at the central value (coded form 0). The interaction between DS-C and FS-FR had the most impact on the permeation flux (Figure 8a). As shown (Figure 8a), an increase in DS-C and a decrease in FS-FR increased the permeation flux. This result can be explained by the dependence of flux on drawing solution concentration. The DS-C provides the osmotic pressure gradient that draws water from the FS into the DS. Consequently, the higher the DS-C, the higher the permeate flux. At the same time, because the FO process is a dilution process, sufficient contact must be established between the two solutions (DS and FS) to drive the movement of water molecules from FS into DS. Lower FS-FR, therefore, provides enough contact time for this movement to occur [47,48].

Chloride ion enrichment (Figure 8b) increased as the DS-C and FS-FR increased. This is the backward flow of Cl^−^ from the draw solution into the FS, driven by the concentration difference between the DS and FS, and is referred to as reverse solute diffusion (RSD). With an increase in the draw solution concentration (20–50 g/L NaCl), more Cl^−^ would be made available in the DS with no corresponding increase in the FS. The difference in concentration, therefore, causes more backward movement of the ions. In addition, the univalent nature of the Cl^−^ ion facilitates its movement across the membrane. The increase in Cl^−^ enrichment with a corresponding increase in FS-FR may be due to the association of high FRs with the creation of turbulence and the bulk solution–membrane interface, which leads to backward diffusion of solutes from the solution–membrane interface into the bulk solution [25]. When this occurs, the Cl^−^ concentration at the FS-membrane boundary decreases. The lower the Cl^−^ concentration at the solution–membrane boundary, the greater the Cl^−^ diffusion from the DS into the FS. Consequently, high FS-FR causes high Cl^−^ enrichment.

In Figure 8, the interactive effects of FS-FR and DS-C on SO_4_^2−^ and CO_3_^2−^ rejection are shown. From Figure 8c, for all values of DS-C, the SO_4_^2−^ rejection efficiency was at its maximum. There was, however, a gradual decline in SO_4_^2−^ rejection efficiency as FS-FR increases. This may be because the rejection efficiency is more dependent on the nature of the ion and membrane properties rather than the flow rates [28]. SO_4_^2−^ is a divalent ion with a hydration radius of 0.379 nm and low diffusion coefficient of 0.32 × 10^−5^ cm^2^/s [49]. These properties inherently facilitate the rejection of SO_4_^2−^, thereby yielding the observed rejection efficiency. Other authors have also made this observation [50]. In Figure 8d, the two factors interact weakly, yielding a maximum CO_3_^2−^ rejection efficiency of 95%. As in the case of SO_4_^2−^ rejection, the divalent nature of CO_3_^2−^ facilitates its rejection.

### 3.4. Numerical Optimisation and Validation

One of the aims of this study was to optimize the operating conditions of the process in order to improve its overall performance and maximize rejection. The numerical optimisation approach was employed to optimise the three operating conditions. This technique explores the entire design space using the designed models to identify the optimal conditions for each factor within the specified range. The model Equations (6)–(9) serve as the objective functions with the three independent variables (response variables) serving as the constraints. Table 7 gives details of the conditions of optimization. All input variables were within range. Permeation flux, SO_4_^2−^ rejection and CO_3_^2−^ rejection were maximized whereas Cl^−^ enrichment was minimized.

**Figure 9:**
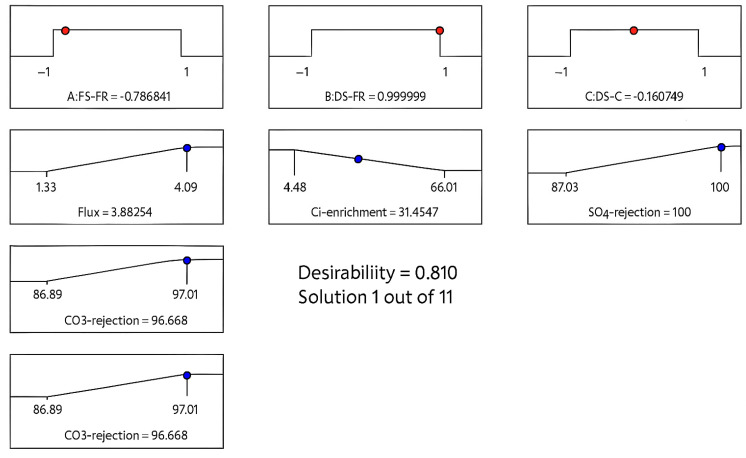
Optimum operating conditions yielding a desirability of 81%, flux of 3.9 L/m^2^h, SO_4_^2−^ rejection of 100%, CO_3_^2−^ rejection of 97%, and Cl^−^ enrichment of 31%.

The result of the optimisation is shown in Figure 9. The ramp graphs show the optimal operating conditions and the resulting desirability. For a desirability of 81% and FS-FR of 9.2 L/h, DS-FR of 9.4 L/h and DS-C of 32.6 g/L (all corresponding to the coded values in the ramp plot), a permeation flux of 3.9 L/m^2^h, SO_4_^2−^ rejection of 100%, CO_3_^2−^ rejection of 97% and Cl^−^ enrichment of 31% would be achieved. These results were further validated with three confirmatory runs.

#### 3.4.1. Confirmatory and Validation

**Figure 10:**
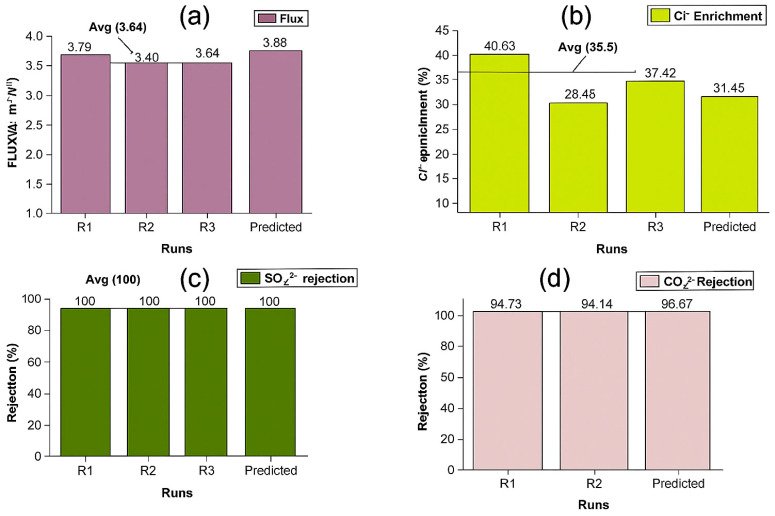
Values obtained for confirmatory runs; (**a**): Permeation flux; (**b**): Cl^−^ enrichment; (**c**): SO_4_^2−^ rejection; (**d**): CO_3_^2−^ rejection.

Figure 10 shows the results of the confirmatory runs. These runs further validated the accuracy of the predictive models. On average, apart from the Cl^−^ enrichment, which shows a wide variation (35.5 ± 5.15%), all other responses (flux: 3.64 ± 0.13; SO_4_^2−^ rejection: 100%; CO_3_^2−^ rejection: 94.59 ± 0.32%) were in close agreement with the predicted values.

Table 8 shows the water chemistry before and after treatment (confirmatory runs) of the raw effluent. The average values of the parameters studied are presented. Apart from Cl^−^, the rejection efficiency of the FO process was good. Size exclusion and electrostatic effects play a significant role in a membrane’s ability to reject ions. This may have accounted for the strong rejection of SO_4_^2−^ and CO_3_^2−^, which are divalent anions. Above the isoelectric point of the FO membrane (which is at a pH of 4), the membrane becomes slightly negatively charged, inducing some resistance to the permeation of SO_4_^2−^ and CO_3_^2−^ [51,52].

There was a total rejection of SO_4_^2−^. This could be due to its large hydration radius (0.379 nm) and low diffusion coefficient (0.32 × 10^−5^ cm^2^/s) [46,47], which may have contributed to its rejection. The rejection of Cl^−^ is severely limited by reverse diffusion from the DS into the FS. It should be noted, however, that the CTA membrane has a proven Cl^−^ rejection efficiency of 90–95% [53,54]. A reduction in conductivity is accompanied by a decrease in the concentration of free ions in the system, which defines electrical conductivity. A water pH of 6.5–8.5 is highly desirable. For pH values below or above this range, water becomes corrosive [55].

#### 3.4.2. FO Membrane Cleaning Efficiency—Flux Recoverability

Pretreatment processes play vital roles in mitigating membrane fouling and achieving efficient use of energy. Pretreatment is the preliminary treatment given to wastewater before the application of membrane process. Depending on the nature of the feed, pretreatment may be very simple or very rigorous. Pretreatments basically precondition wastewater for further treatment my changing the physical, chemical, or biological properties of the wastewater [56].

**Figure 11:**
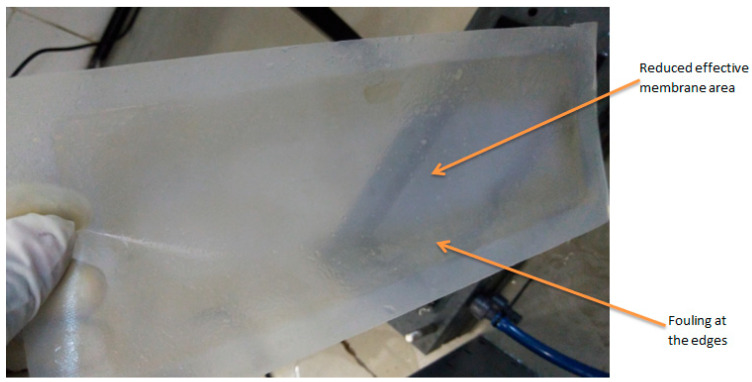
Fouled FO membrane showing reduced effective membrane area.

**Figure 12:**
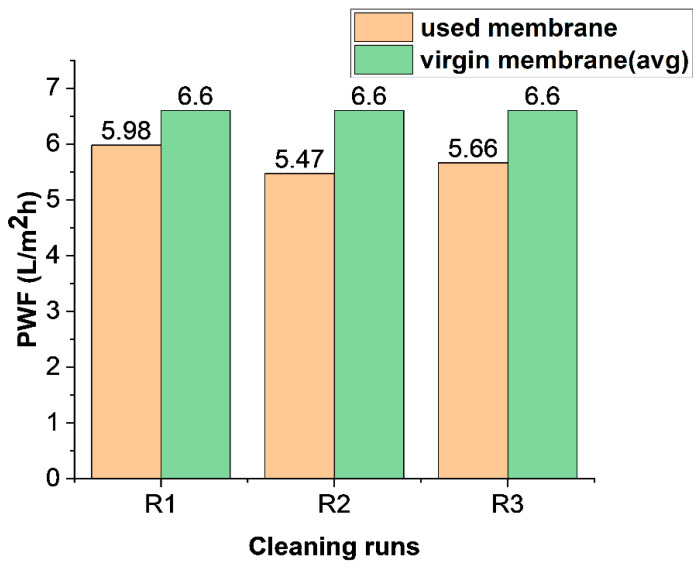
FO membrane flux recovery after membrane cleaning.

The fouled FO membrane (Figure 11) was cleaned using both physical cleaning and osmotic backwashing to remove accumulated dirt and slime from its surface. The membrane’s pure water flux (PWF) after cleaning was compared with that of the virgin membrane. Figure 12 shows the extent of flux recoverability of the FO membrane. On average, the recovered permeation flux was 86.01 ± 2.66%. This indicates a satisfactory result for membrane cleaning. In FO, because external hydraulic pressure is not applied, membrane cleaning readily recovers the flux lost due to fouling. Other studies have shown a slightly higher value for FO flux recovery [57]. However, these membranes were used for fewer hours than the cumulative 30 h, as in this study.

#### 3.4.3. Reverse Solute Diffusion (RSD)

**Figure 13:**
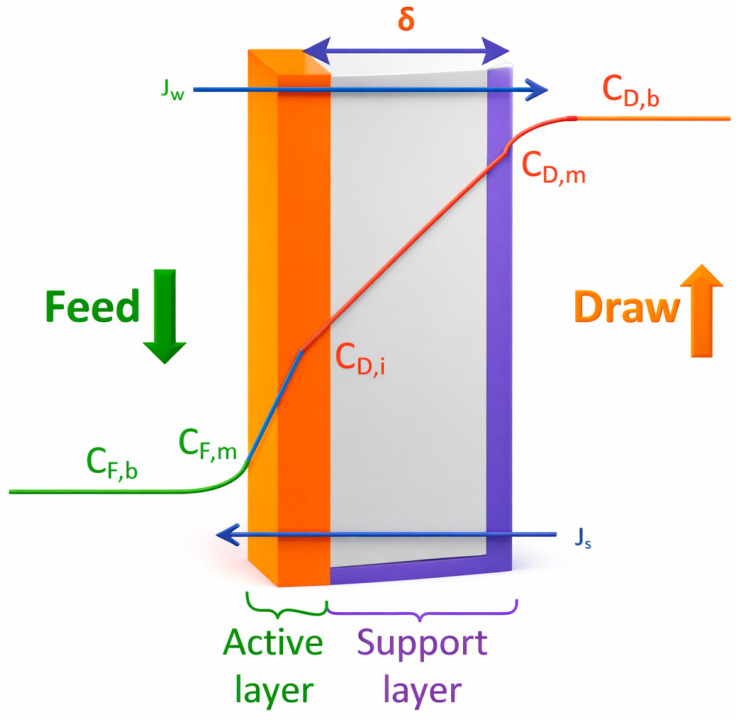
Schematic diagram of the leakage of solutes from the DS into the FS. Modified from Roest [36].

In the FO process, water molecules move into the DS across the semipermeable membrane under osmotic pressure, some of the draw solutes also diffuse from the DS into the FS under the influence of concentration gradient. The forward movement of water (water flux) and the backwards diffusion of solute (RSF) are inevitable due to the system’s tendency to reduce the concentration difference between the FS and the DS. The RSD is undesirable, as it tends to contaminate the FS, leads to loss of draw solute as well as causes a reduction in osmotic pressure of the system [24,58]. Figure 13 shows a schematic diagram of the leakage of solutes from the DS into the FS. During this process, the draw solute first diffuses into the support layer of the asymmetric membrane (FO mode). This diffusion continues until it reaches the interface between the support layer and the active layer, where the draw solutes spread within the active layer before finally diffusing into the FS [59].

Layer by layer considerations for water transport and reverse solute diffusion in FO membrane Equations (13) and (14) form the bases for transport across the FO membrane. From Equation (13), an expression for reverse solute across the active layer of the membrane (as shown in Figure 13) can be written as follows:

(13) $${-J}_{s}=-B\left(C_{D,i}-\;C_{F,m}\right)$$

where ${-J}_{s}$ is the reverse solute flux, B is the solute permeability coefficient, $C_{D,i}$ is the concentration of solute at the porous support layer–active layer interface, and $C_{F,m}$ is the concentration of solute at the feed–membrane interface.

The salt flux across the porous support is the combination of the diffusive component, driven by the salt concentration gradient, and the convective component, resulting from the movement of water through the membrane [45]. This implies that (14):

(14) $$-J_{s\;}=-D^{S}\;\frac{dc\left(x\right)}{dx}+\;J_{w\;}c(x)$$

where *D^S^* is the effective solute diffusion coefficient in the support layer (m^2^/s) and *c*(*x*) is the solute concentration at position *x* within the membrane. The effective solute diffusion coefficient is related to the bulk diffusion coefficient (*D*) by the porosity (ε) and tortuosity (τ) of the support layer according to Equation (15) [59].

(15) $$D^{S}=\frac{D\varepsilon }{\tau }$$

At steady state conditions, the RSF within the active layer and the porous layer should be equal. Mathematically, this yields the following Equation (16):

(16) $$\frac{dc\left(x\right)}{dx}-\;\frac{J_{w\;}}{D^{S}}\;c\left(x\right)=\;\frac{B}{D^{S}}\;(c_{D,i}-\;c_{F,m})$$

Integrating the above equation (Equation (16)), from x = 0 (porous layer–draw solution interface) to x = −δ (porous layer–active layer interface), it follows that (17):

(17) $$c_{D,i}=C_{D,m\;}exp\left(\frac{J_{w\;}S}{D}\right)\;+\;\frac{B}{J_{w\;}}\;\left(c_{D,i}-\;c_{F,m}\right)\left(\exp\left(\frac{J_{w}}{D}\right)-1\right)\;$$

where $C_{D,m\;}$ is the solute concentration at the porous support layer–draw solution interface, *S* is the structural parameter (m).

So far, the Equation (18) have only considered the dilutive ICP and the effective water flux. It is, however, important to account for the concentrative and dilutive ECP. During the FO process, solutes from the feed are retained by the membrane, thereby leading to the accumulation of solutes at the membrane active surface, causing ECP in the boundary layer. As such, taking into consideration concentrative and dilutive ECP,

(18) $$-J_{s\;}=-D^{S}\;\frac{dc\left(z\right)}{dz}+J_{w\;}c(z)$$

where c(z) is the concentration of solute at the position *z* within the boundary layer. At steady state conditions, the solute flux within the ECP boundary layer and the solute flux within the active layer are the same. Integrating from *z* = 0 (ECP boundary layer, where the solute concentration is $c_{F,m}$) to *z* = −δ (where the solute concentration is $C_{F,b\;}$) results (19) in the following:

(19) $$c_{F,m}=C_{F,b\;}exp\left(\frac{J_{w\;}}{k}\right)-\;\frac{B}{J_{w\;}}\;\left(c_{D,i}-\;c_{F,m}\right)\left(1-\exp\left(\frac{J_{w}}{k}\right)\right)$$

where $C_{F,b}$ is the solute concentration in the bulk of the feed solution and *k* is the boundary layer mass transfer coefficient (m/s). To finally write the general expression for water flux and RSF, the following assumptions were made [60].

Osmotic pressure is linearly proportional to the salt concentration, hence ∆π = ∆c.

ECP in the DS is negligible because the support layer thickness is relatively large, thereby dominating the concentration polarization; hence *π_D_*_,*S*_ ≈ *π_D_*_,*b*_.

Substituting Equations (18) and (19) into Equations (20) and (21) yields the following expressions for $J_{w}$ and $J_{s}$, respectively:

(20) $$J_{w}=A\left(\frac{\pi_{D,b}\exp\left({-J}_{w}k\right)-\;\pi_{F,b}\exp\left(\frac{J_{w}}{k}\right)\;}{1+\;\frac{B}{J_{w\;}}\;\left(\exp\left(\frac{J_{w}}{k}\right)-\exp\left(-\;J_{w}K\right)\right)}\right)\;$$

(21) $$J_{s}=B\left(\frac{C_{D,b}\exp\left({-J}_{w}k\right)-C_{F,b}\exp\left(\frac{J_{w}}{k}\right)\;}{1+\frac{B}{J_{w\;}}\;\left(\exp\left(\frac{J_{w}}{k}\right)-\exp\left(-J_{w}K\right)\right)}\right)$$

Application of Equations (17) and (18) are for the determination and simulation of water flux and reverse solute flux in membrane production and fabrication.

## 4. Conclusions

This study successfully optimised key forward osmosis (FO) process variables—feed solution flow rate, draw solution flow rate, and draw solution concentration—using a Box–Behnken design within the response surface methodology framework for the desalination of oil refinery effluent. The developed models for permeation flux, chloride enrichment, sulphate rejection, and carbonate rejection were statistically significant based on ANOVA, confirming the reliability of the optimisation approach. Draw solution concentration emerged as the most influential factor, particularly affecting flux and chloride migration. The optimum conditions (FS-FR: 9.2 L/h; DS-FR: 9.4 L/h; DS-C: 32.6 g/L) produced experimental values—flux: 3.64 ± 0.13 L/m^2^·h; Cl^−^ enrichment: 35.5 ± 5.15%; SO_4_^2−^ rejection: 100%; CO_3_^2−^ rejection: 94.59 ± 0.32%—that closely matched the model predictions, validating the optimisation results. While the optimisation objectives of this study were achieved, we acknowledge that FO treatment of industrial effluent requires longer operational assessment. Future work will therefore involve extended trials (≥24 h), cyclic or repeated-run operation, and pilot-scale testing to evaluate long-term fouling behaviour, flux sustainability, and the progressive impacts of reverse solute diffusion under practical conditions. Such studies will broaden the applicability of FO and support the development of robust operating strategies for diverse wastewater and desalination applications. Additional future work will explore quantifying mechanisms through zeta potential measurements as a function of pH and ionic strength, pore size characterization via porometry and atomic force microscopy (AFM), fixed-charge density estimation, and benchmarking against theoretical models such as Spiegler–Kedem and Steric Hindrance Pore with Donnan. This future work seeks to explicitly evaluate Donnan partitioning and its coupling with internal concentration polarisation. Importantly, this optimisation study was designed to identify the best operating point within the defined experimental design space, acknowledging that the selected parameter ranges were based on practical hydrodynamic and operational constraints. Future work will therefore focus on expanding the parameter space, particularly at higher feed-solution flow rates (FS-FR), to determine whether additional performance gains can be realised beyond the current operational limits. Such extended exploration will provide deeper insight into the FO system’s full optimisation potential.

The study provides an optimised operating window for forward osmosis in refinery effluent desalination, supported by statistically validated modelling and an improved understanding of solute behaviour. While the present investigation focuses on identifying optimal conditions, future work will expand the scope by achieving full quantitative mass-balance closure to better resolve solute-flux pathways, conducting long-term operational assessments to evaluate membrane stability and fouling dynamics, and undertaking deeper mechanistic membrane characterisation to elucidate structure–performance relationships. These aspects, though beyond the current optimisation-driven framework, will strengthen process reliability and enable more comprehensive system-scale evaluations in subsequent studies.

## Figures and Tables

**Table 1:** Characteristics of feed solution.

Component	Average
SO_4_^2−^ (mg/L)	200 ± 43
CO_3_^2−^ (mg/L)	20 ± 1
Cl^−^ (mg/L)	439 ± 98
pH	6
Conductivity (mS/cm)	4.23 ± 0.3

**Table 2:** Box–Behnken design matrix, experimental results and predicted results.

	Factor A (Coded)	Factor B (Coded)	Factor C (Coded)	R_1_ (Flux), L/m^2^h		R_2_ Cl^−^ Enrich (%)		R_3_ SO_4_^2−^ Rejec (%)		R_4_ CO_3_^2−^ Rejec (%)	
RUN	FS-FR (L/h)	DS-FR (L/h)	DS-C (g/L)	Actual	Predicted	Actual	Predicted	Actual	Predicted	Actual	Predicted
1	1	0	−1	1.72	1.74	4.48	4.32	88.91	89.59	89.23	89.36
2	1	1	0	2.59	2.63	20.29	20.52	87.03	87.17	92.49	92.92
3	0	0	0	3.05	3.21	27.9	27.01	96.94	98.48	95.01	94.95
4	0	1	1	3.03	3.06	52.11	52.74	100	98.33	93.28	93.39
5	−1	0	1	4.01	3.96	39.33	39.98	99.05	98.74	94.03	94.36
6	−1	1	0	4.09	4.1	35.01	33.79	99.96	100.42	96.08	96.23
7	1	−1	0	3.04	3.01	41.08	42.8	99.22	98.39	92.29	92.5
8	1	0	1	2.98	2.9	66.01	65.2	95.97	95.97	93.89	93.12
9	0	−1	1	3.71	3.81	63.09	62.62	100	102.04	97.01	97.34
10	0	−1	−1	1.33	1.34	29.33	27.82	100	98.62	86.89	87.13
11	−1	−1	0	3.19	3.13	31.01	31.27	97.13	96.61	91.73	91.66
12	−1	0	−1	2.23	2.27	29.98	31.28	97.92	98.29	90.99	90.59
13	0	0	0	3.18	3.21	26.22	27.01	99.96	98.48	95.28	94.95
14	0	0	0	3.36	3.21	27.9	27.01	97.91	98.48	95.28	94.95
15	0	1	−1	2.74	2.68	17.58	17.94	94.53	94.92	96.04	96.07

**Table 3:** ANOVA of reduced quadratic model for permeation flux.

Source	Sum of Squares	df	Mean Square	F-Value	*p*-Value	
Model	3776.56	7	539.51	300.1	<0.0001	significant
A-FS-FR	1.51	1	1.51	0.8372	0.3906	
B-DS-FR	195.23	1	195.23	108.6	<0.0001	
C-DS-C	2421.04	1	2421.04	1346.71	<0.0001	
AB	153.64	1	153.64	85.46	<0.0001	
AC	680.69	1	680.69	378.63	<0.0001	
B^2^	95.99	1	95.99	53.39	0.0002	
C^2^	248.91	1	248.91	138.45	<0.0001	
Residual	12.58	7	1.8			
Lack of Fit	10.7	5	2.14	2.28	0.3329	not significant
Pure Error	1.88	2	0.9408			
Cor Total	3789.15	14				
R^2^ (0.9967)	Adjusted R^2^ (0.9934)	Predicted R^2^ (0.9759)	Adeq Precision (62.1769)	Std. Dev. (1.34)	Mean (34.09)	

**Table 4:** ANOVA of reduced quadratic model for Cl¯ Enrichment.

Source	Sum of Squares	df	Mean Square	F-Value	*p*-Value	
Model	1721.24	7	245.89	284.48	<0.0001	significant
A-FS-FR	54.44	1	54.44	62.89	0.0006	
B-DS-FR	46.51	1	46.51	53.81	<0.0001	
C-DS-C	1353.56	1	1353.56	1565.99	<0.0001	
AB	124.77	1	124.77	144.35	<0.0001	
AC	98.66	1	98.66	114.14	<0.0001	
B^2^	35.35	1	35.35	40.90	0.0002	
C^2^	8.29	1	8.29	9.59	<0.0001	
Residual	6.05	7	0.8643			
Lack of Fit	5.55	5	1.11	4.48	0.1926	not significant
Pure Error	0.4963	2	0.2481			
Cor Total	3789.15	14				
R^2^ (0.9967)	Adjusted R^2^ (0.9934)	Predicted R^2^ (0.9759)	Adeq Precision (62.1769)	Std. Dev. (1.34)	Mean (34.09)	

**Table 5:** ANOVA of reduced quadratic model for SO_4_^2−^ Rejection.

Source	Sum of Squares	df	Mean Square	F-Value	*p*-Value	
Model	211.6	6	35.27	17.91	0.0003	significant
A-FS-FR	65.72	1	65.72	33.38	0.0004	
B-DS-FR	27.49	1	27.49	13.96	0.0057	
C-DS-C	23.32	1	23.32	11.85	0.0088	
AB	56.4	1	56.4	28.65	0.0007	
AC	8.79	1	8.79	4.47	0.0675	
A^2^	29.87	1	29.87	15.17	0.0046	
Residual	15.75	8	1.97			
Lack of Fit	10.99	6	1.83	0.7708	0.6598	not significant
Pure Error	4.75	2	2.38			
Cor Total	227.35	14				
R^2^ (0.9307)	Adjusted R^2^ (0.8788)	Predicted R^2^ (0.8047)	Adeq Precision (15.5084)	Std. Dev. (1.40)	Mean (96.97)	

**Table 6:** ANOVA of reduced quadratic model for CO_3_^2−^ Rejection.

Source	Sum of Squares	df	Mean Square	F-Value	*p*-Value	
Model	106.91	8	13.36	92.36	<0.0001	significant
A-FS-FR	3.04	1	3.04	21	0.0038	
B-DS-FR	12.43	1	12.43	85.88	<0.0001	
C-DS-C	28.35	1	28.35	195.94	<0.0001	
AB	4.31	1	4.31	29.76	0.0016	
AC	0.6561	1	0.6561	4.53	0.0773	
BC	41.47	1	41.47	286.64	<0.0001	
A^2^	9.83	1	9.83	67.92	0.0002	
C^2^	8.02	1	8.02	55.4	0.0003	
Residual	0.8681	6	0.1447			
Lack of Fit	0.8195	4	0.2049	8.43	0.1088	not significant
Pure Error	0.0486	2	0.0243			
Cor Total	107.78	14				
R^2^ (0.9919)	Adjusted R^2^ (0.9812)	Predicted R^2^ (0.9329)	Adeq Precision (34.6355)	Std. Dev. (0.3804)	Mean (93.30)	

**Table 7:** Conditions for optimization.

Variable	Lower Limit	Higher Limit	Goal
FS-FR (L/h)	7.5	9.4	Within range
DS-FR (L/h)	7.5	9.4	Within range
DS-C (g/L)	20	50	Within range
Permeation flux (L/m^2^h)	1.34	4.10	maximize
Cl^−^ enrichment (%)	4.48	66.01	minimize
SO_4_^2−^ rejection (%)	87.03	100	maximize
CO_3_^2−^ rejection (%)	86.89	97.01	maximize

**Table 8:** Water chemistry before and after application of FO.

Parameter	Value (Avg)	
	Raw	Treated
pH	9.52 ± 0.37	8.4 ± 0.51
Conductivity (mS/cm)	13.02 ± 0.47	1.31 ± 0.41
Cl^−^ concentration (mg/L)	719.62 ± 4.38	Limited *
SO_4_^2−^ concentration (mg/L)	870 ± 15.52	0
CO_3_^2−^ concentration (mg/L)	213 ± 5.6	11.15 ± 0.66

* further explanations given below.

## Data Availability

The original contributions presented in this study are included in the article. Further inquiries can be directed to the corresponding author.

## Abbreviations

BBD	Box–Behnken design
CCD	Central composite design
C_f_	Final concentration
C_0_	Initial concentration
CTA	Cellulose triacetate
D_f_	Dilution factor
DI	Deionized
DS	Draw solution
DS-C	Draw solution concentration
DS-FR	Draw solution flow rate
FO	Forward osmosis
FS	Feed solution
FS-FR	Feed solution flow rate
NF	Nanofiltration
OFAT	One-factor-at-a-time
RO	Reverse osmosis
RSD	Reverse salt diffusion
RSM	Response surface methodology
UF	Ultrafiltration
V_f_	Final volume
V_p_	Volume of permeate
